# Interventions co-designed by healthcare providers and clients for improving therapeutic relationships in maternal and child healthcare: a pilot study using human centered design in rural Tanzania

**DOI:** 10.1186/s12912-023-01472-w

**Published:** 2023-09-14

**Authors:** Kahabi Isangula, Eunice S. Pallangyo, Eunice Ndirangu-Mugo

**Affiliations:** 1 School of Nursing and Midwifery, The Aga Khan University, Dar Es Salaam, Tanzania; 2 School of Nursing and Midwifery, The Aga Khan University, Nairobi, Kenya

**Keywords:** Human-centred design, Nurse-client relationship, Patient-provider relationship, Rural, Tanzania, Africa

## Abstract

**Background:**

Research shows that poor provider–client interactions in maternity and child health (MCH) continue to affect health outcomes, service uptake, continuity of care, and trust in formal healthcare systems.

**Objective:**

The study’s objective was to jointly create a prototype intervention package for enhancing nurse-client relationships using human centered design (HCD) approach.

**Methods:**

A five-step HCD methodology was used: (1) Community-driven discovery through qualitative descriptive research methods using 9 focus groups with nurses and clients and 12 key informant interviews with MCH administrators; (2) consultative ideation and co-creation meetings with 10 nurses, 10 clients, and 10 administrators to co-design a rough prototype model; (3) rough prototype validation through qualitative insight gathering using 6 FGDs with nurses and clients; (4) refinement and adaptation meetings with 14 nurses, 14 clients and 12 administrators; and (5) documentation and sharing of lessons learnt.

**Results:**

According to the community-driven research, poor service, a lack of concern, poor communication, a bad attitude, and unhappiness at work are the nurse factors that affect the relationships between nurses and their patients. Non-compliance with procedures, unfavorable attitudes, poor communication, low education, poverty, and faith in conventional healers were among the client-related factors. Inadequate funding, bad management techniques, improper policy execution, and a lack of an independent institution for handling complaints are the health system factors that affect nurse-client relationships. In response, three ideation and co-creating meetings resulted in 24 interventions. Seven (7) of these were rated as more acceptable and feasible in the local context and formed a rough prototype. During validation, there were some disagreements on the feasibility of curriculum and resource-related interventions. Refinement meetings resulted in a final prototype including four interventions: (i) promotion of patient-centred care; (ii) awards and recognition for nurses; (iii) strengthening complaints mechanisms and (iv) disciplinary measures for abusive nurses and clients. The lessons learnt have been shared through publications and institutional research meetings.

**Conclusions:**

HCD approach provides a novel entry point for providers and clients to examine the problems and design interventions for strengthening their therapeutic relationships in MCH care. Researchers, practitioners, and policy developers are welcome to consider the emerging prototype as it was deemed acceptable and potentially feasible in rural African contexts.

## Contributions to the literature

- The paper presents the step-by-step process of employing a human-centred design to co-develop an intervention package with nurses and clients for strengthening their relationships in maternal and child health clinics in rural Tanzania.
- The paper unpacks the intervention package with the expectation that its implementation may contribute towards addressing the long-standing client dissatisfactions, low service utilization and inadequate service continuity in rural communities.
- The paper offers evidence that human-centred design is a useful approach to exploring complex problems and welcomes researchers, practitioners, and policy developers to consider the emerging prototype in strengthening interpersonal relationships in healthcare.

## Introduction

Nurses and midwives are a crucial part of the healthcare workforce and their contribution is indispensable in the delivery of maternity and child health (MCH) services globally. In many parts of the world, nurses and midwives continue to play a crucial part in the provision of MCH care during pregnancy, delivery and postnatal period [1–4]. In low-income African countries, nurses and midwives are among the well-recognized cadre and most trustworthy providers of medical advice and evidence-based knowledge on a variety of health issues, such as family planning, the care of young children, and disease prevention [2–4]. According to published studies, 83-percent of stillbirths, neonatal fatalities, and maternal deaths may be avoided in a health system with sufficiently qualified and adequately staffed nurses and midwives [5–7]. Relatedly, skilled and competent nurses and midwives have been suggested to increase client uptake and continuity of MCH services [8]. The literature also implies that funding interventions that support nurses and midwives may result in a 16-fold return on investment due to better MCH outcomes [9].

Despite the huge contribution of nurses and midwives, there has been challenges within the nurse-client relationships in particular, a recent rise in client dissatisfaction with their technical and interpersonal skills in MCH services [10–19]. Concerns about technical incompetence related to poor reliability, poor skills, inadequate confidentiality, inability to offer assurance, and inadequate patient engagement; behavioural incompetence related to lack of empathy, poor demeanors, and negative attitudes; poor communication skills and language; violation of client rights; and lack of respect continues to negatively impact the potential contribution of midwives and nurses in the MCH care. This partially explains why recent studies link patients’ unhappiness with nurses’ interpersonal and technical skills to their lack of trust in the formal healthcare system, decreased service uptake and continuity, and poor MCH outcomes [19–23]. Isangula ([23] p29) suggests that the [nurse]-client relationship may involve “*a mutual participation of nurses and clients as they interact for therapeutic purposes in a safe and constructive environment that, in time may allow[s] the client to convey highly personal and private matters”.* This means, a sour relationship between the two may reduce the potential contribution of nurses and midwives within MCH care.

There have been numerous initiatives to improve provider–client relationships by resolving patients’ complaints about healthcare professionals, particularly nurses and midwives. In both high- and low-income settings, the importance of healthcare service governance instruments such as customer charters, policies, complaint mechanisms, medical professional bodies and facility governance committees have been highlighted as attempts to improve provider–client relationships [23]. The key issue with these strategies is that it is difficult to determine whether they are helpful. This may somewhat explain why clients frequently rely on political actions, such as firing employees and calling nurses indolent and dishonest [23]. Notably, relationships between clients and nurses become tense and contentious as a result of political mismanagement of the nurse-client issues, which also lowers provider morale and could further deteriorate provider-patient interactions [23–25]. Likewise, competence-based strategies have been put into practice with a focus on enhancing provider communication skills, patient literacy, patient-centred care, patient information seeking and healthcare facility navigation, patient engagement in care, and questioning skills [23]. These initiatives, however, are frequently carried out haphazardly and provide unsatisfactory outcomes. What appears to be forgotten in current approaches is the complexity of nurse-client relationships within MCH care. It is crucial to keep in mind that problems with providers’ poor interpersonal and technical skills, patients’ socioeconomic vulnerability, literacy, and behaviors, and problems with the health system all have an impact on the nurse-client or provider–client relationships on a regular basis [23]. It may be argued that dealing with this complexity requires specialized, contextualized, and creative approaches that put nurses and patients at the frontline in the creation and application of corrective solutions [26].

In order to improve the delivery of high-quality and acceptable MCH care in resource-constrained settings like rural Tanzania through nurses and midwives, new and novel interventions are required rather than copying existing programs that might not be contextually suitable [26–32]. If adopted, a stepwise incremental process from intervention design to effectiveness evaluation could provide flexibility in problem-solving while adopting a standardized process that has the potential to be utilized in a variety of settings. In order to better understand and address the identified challenges, the Aga Khan University (AKU) modified and piloted a human-centered design (HCD) intervention in rural Tanzania. HCD, in the words of Abookire et al. [31], is an innovative approach to problem-solving that leverages insights from the end users of new products and services, and their experiences to co-create solutions that can be fine-tuned iteratively. HCD is thought to provide more satisfaction among end users as well as greater efficiency and teamwork in the process of developing and implementing public health interventions [30–32]. In addition, compared to conventional problem-solving techniques in the fields of healthcare and public health, HCD may lead to solutions that are more effective and long-lasting [31]. Recently, Melles et al. [30] argued that the application of HCD in health care needs to concentrate on growing awareness of the people encountering a specific barrier and their needs and involving them as stakeholders throughout the process. By taking into account how factors interact at various levels and combining individual interests to create communal interests while designing solutions, the HCD approach also adopts a system-wide outlook. In order to contextualize solutions for complex issues affecting interactions in MCH care, Nurses and Midwives and MCH clients were invited to participate in the intervention design and evaluation process. The aim was to jointly develop an intervention package (prototype) for strengthening nurse-client relationships in the rural Tanzanian province of Shinyanga. The new prototype is anticipated to hold great promise for enhancing nurse-client interactions by boosting client satisfaction, MCH service uptake, and service continuity in rural areas.

## Methods

### Study design

There is already a published protocol for this investigation [33]. Briefly, we employed a qualitative descriptive design with focus group discussions (FGDs), key informant interviews (KIIs), and consultative meeting sessions using HCD as an investigative framework. A five-step HCD approach was briefly used to co-create interventions for strengthening nurse-client relationships. HCD is a method for solving problems that is frequently used in business and is currently gaining popularity as a framework for participatory research in healthcare and public health [26–32]. We recognize that other participatory research frameworks do exist, however, HCD *“use systematic inquiry in direct collaboration with those affected by an issue being studied for the purpose of action or change”* ([34] p1). A thorough examination of a variety of participatory research techniques, from those used in business and social science to those used in healthcare, is provided by Vaughn & Jacquez [34]. HCD shares similarities with other participatory research frameworks in that it incorporates end-user feedback at various stages of the study process [34] but differs in that it is an iterative process. HCD employs a series of iterative and frequently nonlinear phases to tailor solutions for complicated challenges by directly incorporating end users in the creation of solutions that are meant for them [26–35]. When developing an intervention plan iteratively, HCD differs from other approaches in that it focuses on using empathy and a thorough grasp of the needs and motives that drive human behaviours [26]. The HCD strategy involves inviting end users to participate in the design and evaluation phases in order to better comprehend, meet, and overcome the difficulties that have been identified. These fundamental HCD principles can be used to improve the design and execution of interventions in complex low-resource contexts where the causes of bad nurse-client relationships may be very different from those in settings with adequate resources.

The purpose of this investigation was to examine and explain nurse-client relationships without testing any preexisting theories, hence a qualitative descriptive technique was appropriate [35]. The qualitative five-step-HCD approach was deemed appropriate to answer the following questions:

- *Design problem:* How nurses and clients can better improve their therapeutic interactions within MCH care rural Tanzania?
- *Research question:* What are the contributors to poor nurse-client relationships in MCH care in rural Tanzania?
- *Design question:* What is the best intervention co-developed by nurses and clients for strengthening nurse-client relationships to address these contributors considering feasibility and acceptability?

The chosen study environment may differ from other contexts in terms of culture, expectations, and resources available within healthcare settings, therefore this approach gave an excellent means to get a comprehensive and extensive understanding of nurse and client views and experiences in that context. With the help of the qualitative descriptive design, we were also able to acknowledge the subjectivity of the nurses and the clients as well as the researcher’s personal experiences with nurse-client interactions and the data collection procedure. Furthermore, we gained knowledge from these experiences of MCH care by listening to the descriptions of the nurses and clients. Additionally, by paying attention to the descriptions of the nurses and clients, we were able to learn from their experiences of providing and receiving MCH care respectively. We then used this knowledge to “influence the design of interventions” through the HCD process and produce research findings that were “specifically relevant to practitioners and policymakers” in order to enhance MCH care [35]. It is critical to stress that the HCD techniques adopted and the qualitative descriptive design were purposefully selected in accordance with our research objective, available resources, and study participants. They are not intended to be prescriptive. This is being aware of the fact that researchers in diverse contexts have either embraced or adapted different HCD phases and methodologies depending on the topic of interest, intended outcomes, resources, and end-users [26–32].

### Study settings

This research was conducted in Shinyanga, a part of Lake Zone that is primarily populated by Bantus. Isangula [23] provides a thorough account of the area. Shinyanga is a region in Tanzania that is classified as having a poor income, to put it briefly. Shinyanga District Council (DC), Shinyanga Municipal Council (MC), Kishapu DC, Msalala DC, Kahama MC, and Kahama DC are the six administrative districts that make up the area. There are two justifications for selecting Shinyanga. First, Sukuma people, who share a variety of sociocultural tradition and practices with little variation, make up the majority of the region’s population. The area was an excellent representation of many other rural areas in Tanzania due to its close cultural uniformity. Second, local data suggest grave concerns about inadequate nurse-client connections in MCH care, despite a number of capacity-building programs [23]. Shinyanga MC was specifically chosen for study because patients have easier access to both the western health care system and traditional care [23].

In order to develop a context-specific intervention approach that can be used in this and other contexts, we acknowledged the value of improving our awareness of the particular obstacles to nurse-client relationships in the Shinyanga region. The framework for the development and evaluation of complex interventions developed by the UK Medical Research Council emphasizes the critical significance that context plays in the modification and use of interventions [36]. Like many other regions of Tanzania and sub-Saharan Africa, the area is home to a diverse spectrum of rural and urban populations, many of which are extremely marginalized and have the ability to have a positive impact on population health. Nevertheless, considering the contextual variations in Tanzania and throughout Africa, the prototype created can offer a useful and excellent model for practicality, feasibility testing and adaptability in diverse testing.

There have been earlier reports of issues with poor nurse-client interactions in MCH in other rural areas of Tanzania and Africa, including Shinyanga [23]. This suggests that, further iterative testing and modification of the prototype during the feasibility study in the study settings, may provide additional information about the prototype’s viability in Tanzania and beyond.

### Study population, sampling and sample size and data collection

**Fig. 1:**
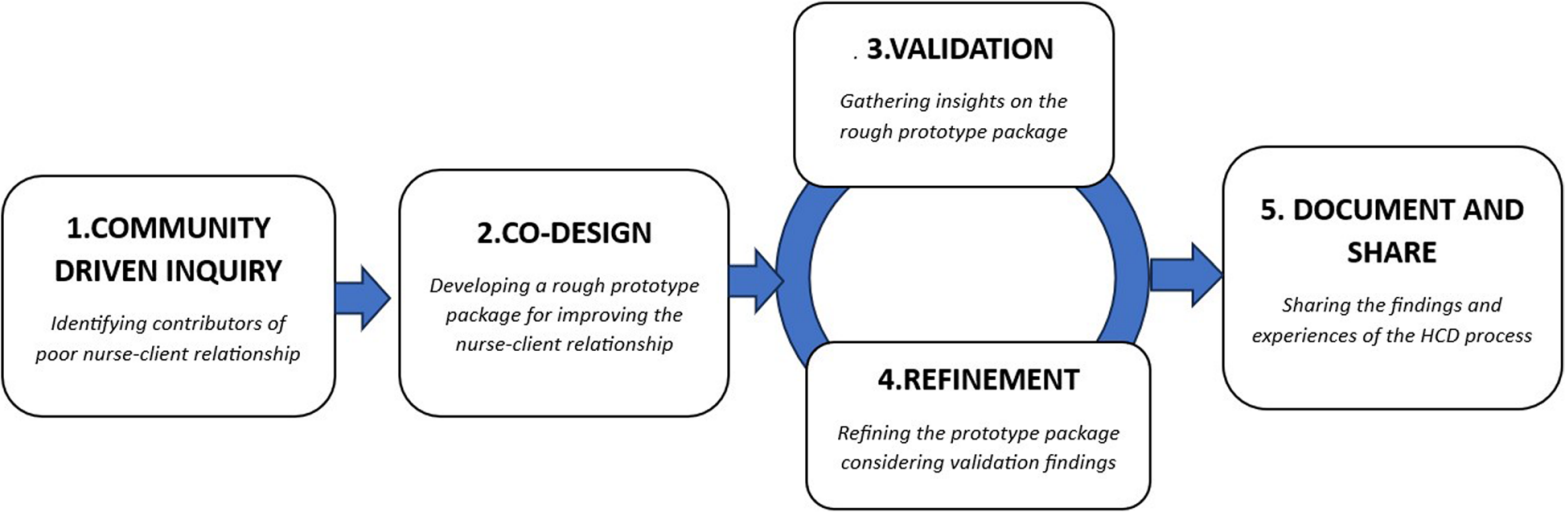
The HCD process

We employed a 5-step HCD process (Fig. 1) each with distinct study population, sampling and sample size and data collection approaches.

#### Step 1. Community-driven discovery inquiry

The procedures for this stage have already been published [33]. In a nutshell, a variety of qualitative research approaches were used to examine both collective and individual viewpoints. A semi-structured interview guide in Swahili was used by the research assistants to conduct about 9 FGDs and 12 KIIs with purposely chosen nurses, midwives, women accessing MCH services, and stakeholders. This sample was sufficient because, according to recent assessments, most qualitative research saturates their data between 9 and 17 interviews [37]. Questions on the current causes of poor nurse-client interactions and the contextual factors, barriers, and facilitators that may have an impact on intervention design, implementation, and sustainability were added in the semi-structured interview guide. Through MCH managers, participants were approached and recruited. Each audio-taped interview was held in a convenient place that had been agreed upon in advance so that respondents might choose a different venue if necessary. Upon arrival, research assistants presented thorough information about the study, got informed consent for an interview and spoke with participants in a semi-structured audio-taped talk for between 45 and 60 min. The findings generated in this step guided Step 2 of the HCD methodology (management and analysis detailed below).

#### Step 2. Consultative co-design meetings

In this stage, a transdisciplinary team of purposively sampled MCH nurses and midwives, clients, administrators, and other relevant stakeholders (30 members) gathered to define the challenges based on discovery findings and design an intervention package (prototype) with the highest potential to improve nurse-client relationships considering acceptability and feasibility. Invitation letters were sent by research assistants to purposively sampled participants with information on the date of the consultative meeting and preselected venue. Guided by the meeting agenda developed by the research team, this process involves 3 consultative meetings: (1) a synthesis meeting to review the qualitative data gathered in step 1 and share insights, experiences, and questions to generate a deeper understanding of the challenges of nurse-client relationships in Shinyanga; (2) an ideation meeting to brainstorm and generate “how might we” questions that facilitated the development of potential ideas for the solution; (3) a prototype and co-creation meeting to evaluate the ideas generated considering pros, cons, and feasibility and develop the initial (rough) prototype model(s) as well as elements crucial to its testing (features, modality, responsible person, etc.). Each meeting was conducted for 4 to 6 hrs, and all key discussion points were documented by research assistants. The findings informed step 3.

#### Step 3. Validation and insight gathering inquiry

This involved gathering insights on the rough prototype in Shinyanga MC on the emerging rough prototype model. The aim was to gather qualitative feedback using guided FGDs (6 sessions) with purposively sampled participants to identify features appealing to both nurses and clients for strengthening their relationship to increase MCH service satisfaction, uptake, and continuity. Nurses and clients were recruited through MCH managers and engaged in 45- to 60-min audio-taped discussions. The findings informed step 4.

#### Step 4. Refinement and adaptation meeting

The design team reconvened for 2 days to evaluate the feedback and rough prototype insights as well as to refine and adapt the prototype. About 10 representatives of the participants for rough prototype testing (insight gathering inquiry) were selected by their peers to join the participants of co-design meetings (making a total of 40 members) in the refinement and adaptation process. About 10 participants of the co-design process who were unable to participate in refinement meetings were replaced with their peers who participated in FGDs and KIIs. The refinement meeting resulted in the final prototype model. The lessons learned in arriving at the final prototype model informed step 5.

#### Step 5. Document and share

The lessons have been synthesized throughout the HCD process and disseminated to local and international stakeholders. These lessons are expected to form the basis for transitioning the intervention package (prototype) to feasibility and definitive testing by researchers as well as influencing policy and practices around therapeutic interactions in MCH care.

It is important to note that for this study, we hired three research assistants who had health science diplomas and trained them in the HCD methodology. To assure readiness for usage during the actual data collection process, the discussion, interview, and consultative meeting guides were pretested in carefully chosen settings and refined. To guarantee the quality of the data, research assistants were closely and encouragingly supervised throughout the stages of data collection and processing.

### Data management and analysis

The FGDs, KIIs, and consultative sessions conducted as part of the HCD process produced a plethora of data. Research assistants simultaneously transcribed and translated data, which was then validated by the research team. For data management and deductive thematic coding, interview transcripts were deidentified, pseudonyms were created for each participant, and the data was then imported into the NVivo 12 program (QSR International). The interview transcripts were subjected to a deductive thematic analysis using a step-by-step process [38]. The study team first looked at the research questions and came up with a few topics that they could all agree on. An analytical matrix of the primary themes and subthemes was produced as a consequence. To create appropriate themes and subthemes in NVivo, individual transcripts and phrases (codes) representing participants’ answers to the researchers’ questions were generated. The research team then utilized a consensus-based methodology to decide whether to include codes that do not fall into the pre-established subthemes and themes; the codes were omitted when they did not, as determined by subjective and objective evaluations, contribute vital value to the study. In order to do interpretive analysis and create reports, the coded data from NVivo were afterwards exported to Microsoft Word (Microsoft Corporation).

## Results

### Participant demographics

The community-driven discovery injury involved 30 nurses (4 FGDs) and 36 clients (5 FGDs) and 12 stakeholders (administrators, NGO representatives and a representative of the Health Facility Governance Committee). Co-design meetings involved an equal number (10) of nurses, clients, and stakeholders. The validation inquiry involved 22 nurses (3 FGDs) and 26 clients (3FGDs). Refinement meetings involved 15 nurses, 15 clients and 10 stakeholders. Females accounted majority of participants (90% discovery), (90% co-design), (96% validation) and (90% refinement participants). On the one hand, most nurses had a higher level of education level (those with college and above were 77% of discovery; 90% co-design; 100% validation and 85% refinement participants) as compared to clients (those with secondary and below, 86% discovery; 70% co-design; 100% validation and 90% refinement participants). On the other hand, most clients had a high number of children as compared to nurses, some of whom had no children (Table 1).

**Table 1:** Participants’ demographics

Category	Community Driven-Discovery				Co-design meetings				Validation Inquiry			Refinement meetings			
	Nurses (%) *n*=30	Clients (%) *n*=36	Admin (%) *n*=12	Total (%) *N*=78	Nurses (%) *n*=10	Clients (%) *n*=10	Admin (%) *n*=10	Total (%) *N*=30	Nurses (%) *n*=22	Clients (%) *n*=26	Total (%) *N*=48	Nurses (%) *n*=15	Clients (%) *n*=15	Admin (%) *n*=10	Total (%) *N*=40
**Gender**															
** Female**	26(87)	36	8 (67)	70(90)	10	10	7(70)	27(90)	20(91)	26(100)	46(96)	13 (87)	14(93)	8(80	35 (87.5)
** Male**	4 (13)	0	4 (33)	8(10)	0	0	3 (30)	3(10)	2(9)	0	2(4)	2(13)	1(7)	2(20)	5 (12.5)
Age															
** <30**	6 (20)	22(61)	0	28(36)	1(10)	4(40)	0	5(17)	5(23)	20(77)	25(52)	7(47)	3(20)	0	10 (25)
** 31-40**	14(46)	13(36)	3(25)	30(38)	5 (50)	6(60)	1(10)	12(40)	6(27)	4(15)	12(25)	6(40)	8(53)	1(10)	15(37.5)
** 41-50**	5(17)	0	8(67)	13(17)	3(30)	0	7(10)	10(33)	6(27)	1(4)	7(15)	2(13)	4(27)	8(80)	14(35)
** >50**	5(17)	1(3)	1(8)	7(9)	1(10)	0	2(20)	3(10)	5(23)	1(4)	4(8)	0	0	1(10)	1(2.5)
Education															
** None**	0	5(14)	0	5(6)	0	1(10)	0	1(3)	0	0	0	0	3(20)	0	3(7.5)
** Primary**	1(3)	17(47)	0	18(23)	0	3(30)	0	3(10)	0	16(62)	16(34)	1(7)	9(60)	0	10(25)
** Secondary**	6(20)	9(25)	1(8)	16(21)	1(10)	3(30)	1(10)	5(17)	0	10(38)	10(21)	5(33)	2(13)	0	7(17.5)
** College**	21(70)	4(11)	2(17)	27(35)	6(60)	2(20)	4(40)	12(40)	21(95)	0	21(44)	8(53)	1(7)	8(80)	17(42.5)
** University**	2(7)	1(3)	9(95)	12(15)	3(30)	1(10)	5(50)	9(30)	1(5)	0	1(2)	1(7)	0	2(20)	3 (7.5)
No. of children (Nurses & clients)															
** None**	4(13)	0	NA	NA	1(10)	0	NA	NA	2(9)	0	2(4)	NA	0	NA	0
** 1-2**	20(67)	21(58)			5(50)	5(50)			11(50)	22(85)	33(69)		10(67)		10 (66.7)
** 3-4**	4(13)	8(22)			2(20)	3(30)			9(41)	1(4)	10(21)		3(20)		3 (20%)
** >5**	2(7)	7(20)			2(20)	2(20)			0	3(11)	3(6)		2(13)		2(13.3)
Years of MCH work/leadership (nurses & administrator)															
** <2**	4(13)	NA	1(8)	NA	1(10)	NA	2(20)	NA	6(27)	NA	NA	NA	NA	2(20)	2(20)
** 2-4**	20{673)		2(17)		7(60)		6(60)		9(41)					4(40)	4(40)
** >5**	6(20)		9(95)		2(20)		2(20)		7(32)					4(40)	4(40)

### Findings from the community-driven discovery inquiry

The results of community-driven research have already been published [39]. Succinctly, nurse, client, and health system characteristics were used to heuristically classify the factors influencing nurse-client relationships. Poor reception and hospitality, failure to show concern and care, poor communication, negative attitudes, poor service quality, work unhappiness, and unstable mental health are all nurse factors that can contribute to poor nurse-client relationships. The clients’ “much known” status, tardiness, disregard for rules and instructions, negative attitudes, poor communication, lack of education and awareness, poverty, dissatisfaction with care, faith in alternative healers, and unstable mental health were the client factors that emerged to contribute to poor nurse-client relationships. Furthermore, bad management practices, ineffective policy execution, insufficient resources, and the absence of a separate department or agency for handling complaints all played a role in the bad nurse-client relationships that plagued the healthcare system and healthcare institutions. These results served as the foundation for the subsequent HCD processes, which were the co-design meetings.

### Findings from consultative co-design meetings

The synthesis meeting formed the first series of co-design meetings. Community-driven inquiry findings were presented, and participants examined the findings considering personal insights, experiences, and questions to generate a deeper understanding of the challenges of nurse-client relationships in Shinyanga. The results of the synthesis meeting indicated a broad consensus on the contributors of poor nurse-client relationships with some refinement and addition of the contributors. For instance, the ineffectiveness of suggestion boxes was highlighted by fears of retaliation among clients, the unfriendliness of suggestion boxes and the lack of feedback on how complaints were handed were among the additions.

The ideation meeting involved group discussion to brainstorm and generate “how might we” questions. This facilitated the development of 82 ideas for potential solutions. These ideas were then reorganized by the research team into 24 broad categories each with several activities considering conceptual convergence and similarities between them. A prototype and co-creation meeting brought together participants in three groups to evaluate the ideas generated during the ideation meeting and the emerging categories considering pros, cons, and feasibility as well as elements crucial to its testing (features, modality, responsible person, etc.). Through consensus building, participants in each group rated the 24 categories (and their related activities) considering feasibility (0-10scores) and acceptability (0-10scores) among nurses and clients. The total scores ranged from 58 for disciplinary measures for abusive nurses and clients (highest), followed by 56 for awards and recognition for nurses, 52 for strengthening complaints mechanisms, 49.5 for improving nursing school curriculum, 49.5 for ensuring availability of resources, 49 developing nursing leaders and 48 for promotion of patient-centred care to 32.5 for ensuring availability of mental health services and support for nurses and clients (Lowest) (Table 2). The meeting resolved to consider the seven interventions with the highest scores as part of the ‘rough prototype model’ that was then subjected to the next HCD step.

**Table 2:** Rating of 24 interventions that emerged from the ideation meeting

	***PROPOSED INTERVENTION***	**PROPOSED ACTIVITIES**	***RATING***						
			***ACCEPTABILITY***			***FEASIBILITY***			***TOTAL SCORE***
			***GROUP 1***	***GROUP 2***	***GROUP 3***	***GROUP 1***	***GROUP 2***	***GROUP 3***	
***1***	***Disciplinary measures for nurses and clients***	• *Simple disciplinary measures for bad nurses* • *Community to create bylaws for clients who abuse nurses*	***10***	***10***	***10***	***10***	***8***	***10***	***58***
***2***	***Awards and recognition for good nurses***	• *Establishment of awards for nurses (monthly/yearly) at the level of facility, district, and region* • *Giving letters of congratulations and posting good nurses on hospital notice boards or recognizing them in formal meetings*	***9***	***10***	***10***	***7***	***10***	***10***	***56***
***3***	***Improving complaints mechanisms***	• *Having a separate entity for gathering handling and communicating complaints* • *Improving suggestion boxes by educating clients on how to complain, strengthening privacy and feedback to clients* • *Conducting Exit interviews with clients before they leave the facility* • *Having a performance evaluation desk for nurses*	***10***	***10***	***10***	***8***	***8***	***6***	***52***
***4***	***Improving nursing curriculum***	• *Increasing the number of hours and credits on the communication skills course* • *Adding customer care courses to the nursing curriculum* • *Improving practical learning environment for nurses to practice ways of improving relationships with clients*	***10***	***10***	***10***	***8***	***2.5***	***9***	***49.5***
***5***	***Improving the availability of resources***	• *Improving procurement process for medications out of stock at MSD* • *Increasing availability of medicine and medical equipment* • *Improving healthcare infrastructure* • *Engaging stakeholders in the availability of resources* • *Designing internal income generation activities without burdening the clients*	***9***	***10***	***10***	***8***	***5***	***7.5***	***49.5***
***6***	***Improving the efficiency of nursing leaders***	• *Encouraging leaders to adhere to leadership ethics* • *Conducting regular mentorship to leaders* • *Training on good leadership* • *Punishment for leaders with many complaints*	***8***	***10***	***10***	***6***	***6***	***9***	***49***
***7***	***Improving patient/Client-Centred Care***	• *Emphasis on clients’ rights e.g., the right to choose providers* • *Offering information to clients on deficits at the facility* • *Avoidance of discrimination during care provision* • *Encourage clients to speak freely* • *Engaging clients in treatment decisions* • *Informing clients in advance on the process of care (steps to go through to receive care)* • *Continuous client education on the care they deserve from nurses*	***8***	***8.5***	***9***	***7***	***7***	***8.5***	***48***
***8***	***Improving health service delivery***	• *Using professional meetings to remind nurses of the need to adhere to ethics* • *Encourage equality in nursing care* • *Establishment of service packages for different income groups without conflicting quality* • *Training nurses on different aspects of nursing care*	***9***	***10***	***8***	***8***	***7***	***6***	***48***
***9***	***Improving and dissemination of service delivery guidelines***	• *Improving existing service guidelines* • *Ensuring that service guidelines are disseminated to all nurses*	***8***	***10***	***10***	***5***	***5***	***9***	***47***
***10***	***Improving the implementation of the National Health Policy***	• *Ensuring the availability of resources as promised in the national health policy* • *Strengthening cost recovery mechanisms for groups who receive free care* • *Improving the effectiveness of health facility governance committees* • *Developing and dissemination of clients’ service charters*	***8***	***10***	***9.5***	***5***	***4***	***9***	***45.5***
***11***	***Improving workplace policy and procedures***	• *Improving work policy and procedures* • *Improving shift arrangements by putting good and bad nurses on a similar shift for co-learning* • *Rotating nurses in different departments*	***7***	***10***	***8.5***	***6***	***5***	***8.5***	***45***
***12***	***Community sensitization***	• *Educating community on early healthcare seeking, early clinic attendance, healthcare service delivery process and the importance of and responsibilities of nurses* • *Continued community education through media outlets (TV & Radio), community meetings, and health education sessions at the clinic CHWs* • *Establishing soci al welfare and community education department at the facility* • *Establishing a system for monitoring and controlling of the correctness of health-related social media content*	***8***	***10***	***7***	***8***	***5***	***7***	***45***
***13***	***Continued professional development for nurses***	• *Establishing orientation packages and orienting new employees on customer care* • *Using formal meetings for reminding nurses about nursing ethics* • *Mentorship for less experienced by experienced nurses* • *Putting less experienced nurses on a similar shift with experienced nurses* • *Frequent training on customer care skills, Nursing delivery process, clients’ rights, use of electronic health information systems and mental health (online or face-to-face)*	***7***	***10***	***10***	***5***	***4***	***9***	***45***
***14***	***Improving nursing workforce***	• *Hiring more new nurses* • *Use of nursing volunteers*	***5***	***10***	***10***	***5***	***5***	***9.5***	***44.5***
***15***	***Team building activities for nurses***	• *Design and implement team-building activities that bring together nurses from within and outside facilities* • *Training on team-building skills* • *Team building trips within and outside the facility, district, region, and country*	***8***	***10***	***10***	***4***	***3***	***9***	***44***
***16***	***More research on provider–client relationships***	• *Stakeholders to conduct more research on effective strategies for improving provider–client relationships*	***5***	***10***	***10***	***2***	***10***	***6.5***	***43.5***
***17***	***Use of religious leaders***	• *Inviting religious leaders to train nurses on ethics and loving care*	***2***	***10***	***10***	***1***	***10***	***10***	***43***
***18***	***Dialogue between facilities and communities***	• *Engage health facility committees in coordinating dialogues between facilities and community members* • *Using community meetings to educate communities on the healthcare delivery process*	***5***	***10***	***10***	***5***	***8***	***4.5***	***42.5***
***19***	***Improving salaries and Allowances***	• *Salary increments for nurses* • *Improving allowances for nurses* • *Timely salary payments* • *Establishment of low-interest loans for nurses*	***5***	***10***	***10***	***5***	***3***	***9.5***	***42.5***
***20***	***The motivation of secondary school students to join the nursing cadre***	• *Nursing boards/associations to sensitize and encourage secondary school students to join a nursing cadre* • *Establish nursing carrier guidance in secondary schools* • *Improving enrollment procedures to identify students who are self-motivated to become nurses*	***7***	***10***	***9***	***6***	***2***	***8***	***42***
***21***	***Reducing politicization of nursing cadre***	• *Encouraging political leaders to avoid interfering with nursing and healthcare service delivery by creating false expectations for clients* • *Educating clients and community members on the reality of healthcare services (to avoid being swayed by politicians)*	***5***	***10***	***10***	***5***	***3***	***8.5***	***41.5***
***22***	***Improving the effectiveness of nursing profession Boards***	• *Nursing professional boards to promote their work to nurses (visiting nurses in their facilities or media outlets)* • *Nursing boards to organize frequent seminars and forums at the district, regional and national levels* • *Nursing Boards to approve customer care and communication skills as CPD courses for nurses* • *Nursing Boards to train nurses in customer care and communication skills*	***6***	***10***	***10***	***5***	***2***	***8***	***41***
***23***	***Improving client’s healthcare purchasing power***	• *Roll of universal healthcare insurance* • *Encourage engagement in income-generating activities* • *Training clients on entrepreneurship skills* • *Implementing poverty elimination strategies* • *Customer segmentation- establishing service packages based on the client’s income without conflicting service quality*	***8***	***10***	***5***	***8***	***4***	***5.5***	***40.5***
***24***	***Mental health support for nurses and clients***	• *Development and distribution of Information, Education and Communication materials on mental health* • *Strengthening the linkage of clients and nurses to mental health support entities* • *Establishing mental health support office/ personnel at least at the health centres and hospital level* • *Posting telephone numbers of mental health personnel/entities on hospital noticeboards* • *Training nurses on mental health skills*	***1***	***10***	***8***	***1***	***8***	***4.5***	***32.5***

### Findings from the validation/insight gathering inquiry

The insights gathered through FGDs with nurses and clients who were not involved in the initial HCD steps indicated a broad consensus that the seven interventions are more likely to improve nurse-client relationships. A range of benefits and disadvantages of these interventions were cited. Of note, the benefits of these interventions cited by participants of validation inquiry are largely similar to those cited by participants of community-driven inquiry. The disadvantages of these interventions included fears among some participants that disciplinary measures will reduce work morale among nurses because poor relationships are often rooted in many health system challenges faced by nurses. Relatedly, a few participants suggested that community bylaws for community members will heighten conflicts between nurses and clients. Furthermore, there was a dominant concern that awards and recognitions for nurses are surrounded by favouritism and offered to high-level nurses who are not directly engaged in the provision of MCH care. Likewise, the awards system does not adequately engage nurses and clients in setting the award criterion. Consequently, a call was made for departmental-level awards, the use of meetings to recognize the best performers and more engagement of nurses, clients, and direct supervisors in the selection of nurses who deserve awards and recognition. Some of these issues can be seen in the following quote:

*The intervention about disciplinary measures to nurses is not attractive to me because we have many challenges…. if you say we start implementing disciplinary measures it will reduce our morale when our challenges are not adequately addressed (Nurse, Health Center)*

**Fig. 2:**
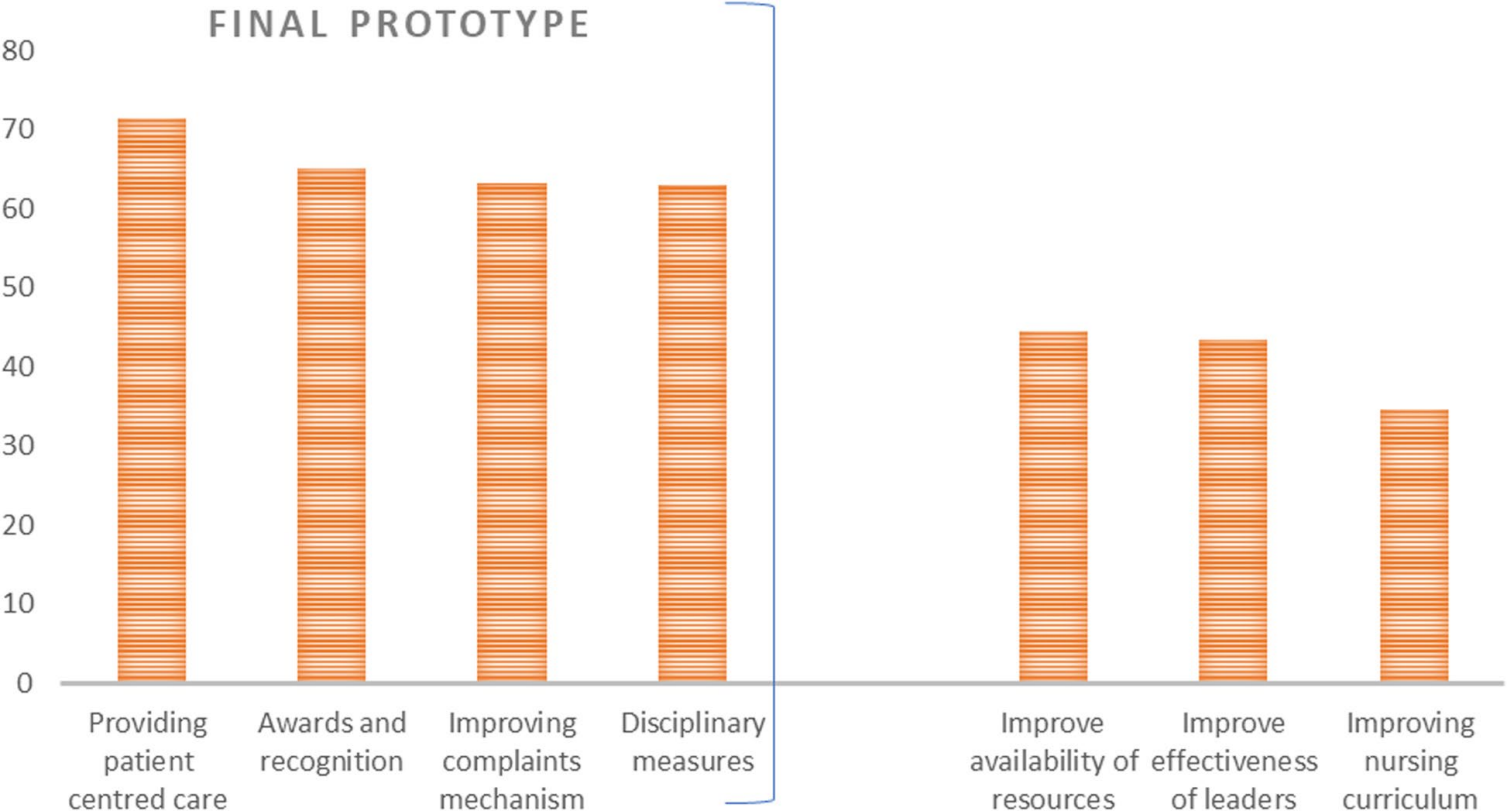
Final prototype model

In general, all the interventions were considered acceptable, however, there were some disagreements on the feasibility of curriculum and resource-related intervention. For instance, restructuring curriculum and resource availability were voted by participants of the validation inquiry to be highly acceptable but they were deemed less feasible in the study contexts because of the time and multistakeholder efforts needed for their successiful implementation. Consequently, the rating of the interventions resulted in the scores portrayed in Fig. 2 below.

### Findings from the rough prototype refinement meeting

Refinement meetings resulted in a final prototype including four interventions: (i) patient-centred care; (ii) awards and recognition for good nurses; (iii) improving complaints mechanisms and (iv) simple disciplinary measures for bad nurses (Fig. 2).

### Documentation and sharing the lessons learnt

Several strategies were employed to document and disseminate the results of this interventional study. First, institutional meetings, journal clubs and networks were used to share the research findings. Second, the final report will be shared with district and regional medical officers, nursing and midwifery councils, the Ministry of Health, and the National Institute for Medical Research for dissemination through government channels. This will ensure that the proposed interventions contribute to practice, policy, and strategic plan discussions at the local and national levels. Third and final, the protocol, results of community-driven inquiry and some parts of the findings of co-design have been published elsewhere [33, 39, 40]. The findings will also be disseminated through international and local conferences.

## Discussion

The purpose of this study was to pilot an HCD approach in the co-designing of a prototype for improving provider–client relationships in MCH care in rural Tanzania. Using this approach, the research team partnered with nurses, clients, and other MCH stakeholders to develop an intervention package for addressing the complex problem of nurse-client relationships in the rural region of Shinyanga. Partnering with MCH administrators, nurses and clients strengthened data source triangulation however, it may have introduced power dynamics in some HCD steps. Power issues have been documented as common in the healthcare sector in previous research [23, 41]. For instance, Isangula [23] highlights issues of power imbalances among providers themselves and between providers and clients in the context of interpersonal relationships in research conducted in a similar setting. In support, Mvuvu [41] observed power dynamics across HCD steps between researchers and providers, healthcare administrators and providers, among providers themselves, and between providers and clients in an urban region of Tanzania. To minimize power dynamics, KIIs with administrators and, FGDs with clients and nurses were conducted separately. During consultative meetings, a requirement was made for the discussion groups to have a client as a chairperson and a research team member was assigned to each group to ensure equal participation.

The principal results of our study are fourfold. First, community-driven inquiry unmasked a range of nurses, clients, and health system drivers of poor nurse-client relationships, detailed elsewhere [39]. The most important and complex contributors of poor nurse-client relationships are the health system and healthcare facility contributors including concerns about inadequate resources, poor management practices, inadequate policy implementation and the absence of an independent department or agency for gathering and management of complaints. The complexity of these contributors is based on the long-standing nature and the massive advocacy, resources and structural changes required to fully address them. Looking across literature both within and outside Tanzania, provider, client, and health system factors have been widely documented as impacting provider and nurse-client relationships in both low- and high-income contexts [10–23]. The problem with previous studies is that a large body of literature has mainly focused on providers’ technical and behavioural competencies as well as health system dysfunctions as primary drivers of poor nurse-client relationships. Consequently, most of the existing interventions -whether governance instruments, competence-based or political- prioritize or lays blames on providers (nurses included) as the prime source of poor provider–client relationships in therapeutic encounters [17–25]. However, our findings recognize clients’ contribution to poor therapeutic relationships. To the best of our knowledge, only a few studies have detailed the role of clients in fueling their negative relationships with their providers. An example is a study by Isangula [23] conducted in a similar setting that recognized clients’ negative behaviours and faith informal medical practices (traditional medicines) are among the key drivers of poor provider–client relationships. This indicates that efforts to address nurse-client relationships need to embrace the complex interaction of nurses, clients, and health system factors contributing to poor therapeutic relationships.

The contributors of poor nurse-client relationships guided the discussions during consultative meetings conducted as part of the co-design process. Rating of the ideas emerging in the co-design process considering feasibility and acceptability resulting in a rough prototype model with awards and recognition for nurses, strengthening complaints mechanisms, improving nursing school curriculum, ensuring availability of resources, developing nursing leaders and promotion of patient centred care scoring higher and forming the ‘rough prototype model. Looking across the literature, some of the proposed interventions within the rough prototype have been previously documented as among the key strategies for strengthening nurse-client relationships [19, 42, 43]. The difference between strategies proposed in previous studies and those emerging from the co-design process of our study is twofold. First, some of the interventions proposed in the current study appear novel and have received weaker attention in previous studies. For instance, awarding better performing nurses, punishing bad nurses and clients, implementing the promises made in the national health policy, use of religious leaders to train nurses on ethics and loving care and mental health support for nurses and clients are some of the novel interventions proposed to strengthen nurse-client relationships. Secondly, each of the proposed interventions in the present study has peculiar activities that are considered of local relevance which may be different from other contexts. For instance, the establishment of awards for nurses (monthly/yearly) at the level of the facility, district, and region and giving letters of congratulations and posting good nurses on hospital notice boards or recognizing them in formal meetings were proposed for awards and recognition intervention. Likewise, having a separate entity for gathering handling and communicating complaints, exit interviews and nursing performance evaluation desks were proposed as activities for improving the complaints mechanism on top of improving the effectiveness of suggestion boxes. This indicates that the use of the HCD approach may result in novel interventions that are more relevant to end users’ contexts.

The seven highly rated interventions were subjected to validation through insight gathering. The findings indicated that all seven interventions were considered acceptable but with various degrees of perceived feasibility. For instance, nursing curriculum and resource-related interventions were considered less feasible because of the time and multistakeholder efforts needed for their successiful implementation [40]. Similar concerns have been demonstrated in previous interventions proposed for improving nurse-client relationships [19, 42–46]. Finally, based on validation findings, subsequent refinements, and rating of the seven interventions, four interventions were selected as the final prototype including (i) patient-centred care; (ii) awards and recognition for good nurses; (iii) improving complaints mechanisms and (iv) simple disciplinary measures for bad nurses. Looking across the existing body of literature in both high- and low-income countries, an emphasis on patient-centred care and improving complaints mechanisms have been previously emphasized for improving provider–client relationships [42–46]. However, as noted above, awards and recognition for good nurses and simple disciplinary measures for bad nurses appear novel and have not been proposed in previous studies to the best of our knowledge. This may have emerged because of the engagement of both nurses and clients from this rural context in the process of co-designing the solutions. As noted above, the strength of the HCD approach is that it is a highly adaptive and creative approach to problem-solving [26–32]. Employing HCD in this study enabled the research team and research participants to conduct a detailed analysis of the contributors to poor nurse-client relationships [33, 39] and ensure that nurses and MCH clients collaborate in the designing of solutions consequently generating a final prototype model considering the perceptions of feasibility and acceptability [33, 39]. We have documented the co-design process and developed the final prototype manual and associated materials to facilitate replication of the intervention in similar or other settings. Specifically, we are now applying for additional funds to test this prototype within Tanzania and across the East African region to determine whether it could apply to a much broader African context.

### Methodological considerations

Key methodological considerations for this study are twofold. First, the HCD intervention uses nurses as exemplars of healthcare providers in the course of co-creation of the prototype model for strengthening interpersonal relationships in MCH care in rural Tanzania [33, 39]. However, we understand that patients interact with a range of other providers within the healthcare system including doctors, laboratory experts, supportive staff, administrators etc. This means if a similar study is conducted with another type of providers and in a different setting for instance urban, it may yield a different prototype model. However, we believe that our prototype model forms a stepping block for addressing the complex problem of poor nurse-client relationships. We welcome researchers and nurses and midwives to consider the interventions proposed in both the rough and final prototype in their attempt to improve nurse-client relationships beyond MCH care. We also encourage future inquiries to extend beyond the nursing cadre and rural context. Second and final, we used purposefully chosen HCD steps to generate an intervention package. These HCD steps may be viewed as divorcing from the traditional prescriptive HCD cycles documented in many high-income countries [47]. This suggests that the efforts to encourage the application of HCD in low-income settings may need to portray HCD as a non-prescriptive cycle or series of steps but a framework that can be adapted depending on the context, participants, intended outcome and resources.

## Conclusions

In conclusion, the HCD approach provides a novel entry point for providers and clients to examine the problems and co-design highly acceptable and contextually feasible interventions for strengthening their therapeutic relationships in MCH care. The results of this pilot study may inform the design of practices, guidelines, and policies to strengthen interpersonal relationships in healthcare settings more broadly. Therefore, we welcome interventionists to consider the emerging prototype as a potential tool for improving interpersonal aspect of MCH care in rural African contexts. Moreover, future implementation teams and researchers can learn from the experience of this HCD intervention to guide solution development for complex healthcare problems.

## Data Availability

The dataset(s) supporting the conclusions of this article are) included within the article. Additional data on the HCD process that are not part of the published article will be available on request from the AKU through the corresponding author (KI). Some data may not publicly available for ethical reasons (ie, information that could compromise the privacy of research participants).
